# Randomized clinical trials in ANCA-associated vasculitis: a systematic analysis of the WHO - International Clinical Trials Registry Platform

**DOI:** 10.1186/s13023-020-01408-6

**Published:** 2020-05-29

**Authors:** Michele Iudici, Xavier Puéchal, Alejandro Brigante, Ignacio Atal, Cem Gabay

**Affiliations:** 1 Division of Rheumatology, Department of Medicine, Geneva University Hospitals, Geneva, Switzerland; 2 National Referral Center for Rare Systemic Autoimmune Diseases, Cochin Hospital, Paris-Descartes University, Paris, France; 3 Sanatorio Güemes, Servicio de Medicina Interna – Reumatología Francisco Acuña de Figueroa 1240, C1180AAD Ciudad Autónoma de Buenos Aires, Argentina; 4 Center for Research and Interdisciplinarity, Université Paris Descartes, Paris, France

**Keywords:** ANCA-vasculitis, Randomized controlled trial, Epidemiology

## Abstract

**Background:**

The analysis of the main features of randomized controlled trials (RCTs) on ANCA-associated vasculitis (AAV) can inform future study design.

**Methods:**

We searched within the International Clinical Trials Registry Platform all registered RCTs on AAV from October 2008 to December 2018. Two reviewers selected studies according to pre-specified eligibility criteria. We retrieved information including countries, funding, design, sample sizes, eligibility criteria, primary outcomes (POs), and treatments.

**Results:**

Among the 40 RCTs identified, 22 (55%) were conducted in Europe, 29 (72,5%) in a single country, 14 (35%) were industry-funded. The median number of patients planned to enrol was 68 (IQR 36–138). Only 28% of RCTs targeted a single vasculitis, and ANCA negative patients were not included in about 40% of studies. Interventions investigated were mainly drugs given to induce (40%) or maintain (32.5%) remission. Eighty-five percent of POs were considered being ‘patient-important’, but discrepancies in definition of disease states, such as remission or relapse were observed. Glucocorticoids use was part of the PO in < 25% of studies. The number of trials targeting a single disease, non-industry funded, incorporating glucocorticoids in PO, as well as the planned sample size increased over time.

**Conclusion:**

Despite the important achievements in the field, a better harmonization of eligibility, and outcome criteria across studies is an important objective to pursue in next future.

## Introduction

Antineutrophil cytoplasmic antibodies (ANCA)-associated vasculitis (AAV) is a rare small vessel vasculitis characterized by multisystemic involvement, need of long-term treatment and potential severe complications [1]. Granulomatosis with polyangiitis (Wegener’s, GPA), microscopic polyangiitis (MPA) and eosinophilic granulomatosis with polyangiitis (EGPA, Churg-Strauss), [2] together with ANCA positive renal-limited vasculitis represent the clinical entities included in this group of diseases. Despite AAV share clinical and serological features, they differ in terms of genetics, pathophysiologic mechanisms, outcomes, main complications, and leading causes of death [1, 3–7]. Performing clinical studies in AAV is therefore challenging, given the rarity, and the complex and heterogeneous clinical presentation of such diseases.

Past years have been characterized by major advances in the treatment of AAV [8, 9]. In 2007, a European League Against Rheumatism (EULAR) task force developed recommendations intended to assist clinical researchers in designing clinical trials in AAV [10, 11].

Nevertheless, it seems that there still exist some issues in trial designs, which need to be addressed. To better delineate the leading features of AAV-randomized clinical trials (RCTs), and possibly identify their main limitations, we planned to perform a systematic analysis of trial protocols registered in international platforms over the last decade.

## Methods

### Search strategy

On 17 January 2019, we searched on the International Clinical Trials Registry Platform (ICTRP) [12] all records from October 2008 through December 2018 of interventional RCTs on AAV. The ICTRP portal provides a single point of access to information about ongoing and completed clinical trials registered around the world. It provides a free searchable database containing the trial registration data sets coming from many data providers including also ClinicalTrials.gov, and EU Clinical Trials Register. We performed a search strategy using the terms ‘ANCA-associated vasculitis’ OR ‘granulomatosis with polyangiitis’ OR ‘microscopic polyangiitis’ OR ‘eosinophilic granulomatosis with polyangiitis’ OR ‘polyangiitis’ OR ‘ANCA’ OR ‘granulomatosis’ OR ‘small-vessel’.

### Eligible criteria, data collection and extraction

All interventional RCTs including patients with AAV (GPA, MPA, EGPA, ANCA positive renal-limited vasculitis) were included. A study was considered to be interventional if participants were assigned receiving one or more therapeutic intervention(s)/treatment(s), as determined by study protocol. We excluded non-randomized studies, fundamental research, diagnostic and cost-effectiveness studies. Two physicians (AB, MI) independently checked the studies against the pre-specified eligibility criteria. Disagreements were discussed by the authors to reach consensus. The same 2 reviewers (AB, MI) independently extracted data from eligible studies by using a standardized form. Consensus was reached by discussion in case of disagreements.

### General characteristics of clinical trials

We assessed study characteristics that included country, start date, funding sources (industry, non-industry), phase of development (phase 0, I, II, III, IV), planned and final sample size, enrollment status (i.e., closed recruitment, recruiting, not yet recruiting, withdrawn), study design (i.e., parallel-arms, cross-over), number of arms, type of intervention (− pharmacologic [biological, non-biological], − non-pharmacologic), type of comparator (placebo, active intervention, usual care, or no intervention). We labeled biotherapy each monoclonal antibody targeting immune cells and/or circulating cytokines. Data on the mechanism(s) of action for each drug under investigation was also collected [13]. A study was considered being industry-funded if the sponsor or one of the collaborators (an organization other than the sponsor providing support for a clinical study) was industry.

### Eligibility criteria and population targeted in trials

The following data on the patients’ characteristics as specified by eligibility criteria was assessed: age [child (< 18 years), adults (18 to 65 years), senior (> 65 years)]; disease; criteria to identify the disease (Chapel-Hill nomenclature [14] or revised Chapel-Hill nomenclature [2], ACR criteria [15, 16], clinical diagnosis); autoantibody specificity (anti-proteinase3 – anti-PR3; anti-myeloperoxydase – anti-MPO; ANCA pattern in immunofluorescence); disease status (newly diagnosed or prevalent patients).

### Evaluation and classification of outcomes

For each RCT, we collected primary outcomes and we assessed the type of outcome (safety, efficacy). All outcomes were then independently classified by 2 of the authors (MI, XP) as Patient-Important Outcomes (PIO), or Surrogate Outcomes (SO) according to previous works on this topic [17, 18]. Consensus was reached by discussion by the 2 authors, and in case of disagreement final classification was discussed with a third investigator. We classified PIO as measures that directly impact quality of life such as major morbid events (e.g. death, end-stage renal disease) or minor morbid events (e.g. minor disease flare, pain and functional status); SOs were classified as measures that may indicate disease progression and increased risk for patient-important outcomes, or assessed response to physiological or laboratory tests without direct tangible effects on patients (e.g. ANCA titer, increased blood cholesterol level, etc.) [17, 18]. Finally, we recorded the number of studies with at least one PIO as primary outcome and if the dose/use of glucocorticoids (GC) was included in primary outcome.

### Data analysis

The analysis was descriptive. Continuous variables were expressed as median [interquartile range], and categorical variables were described with frequencies and percentages.

## Results

### General characteristics of trials

**Fig. 1:**
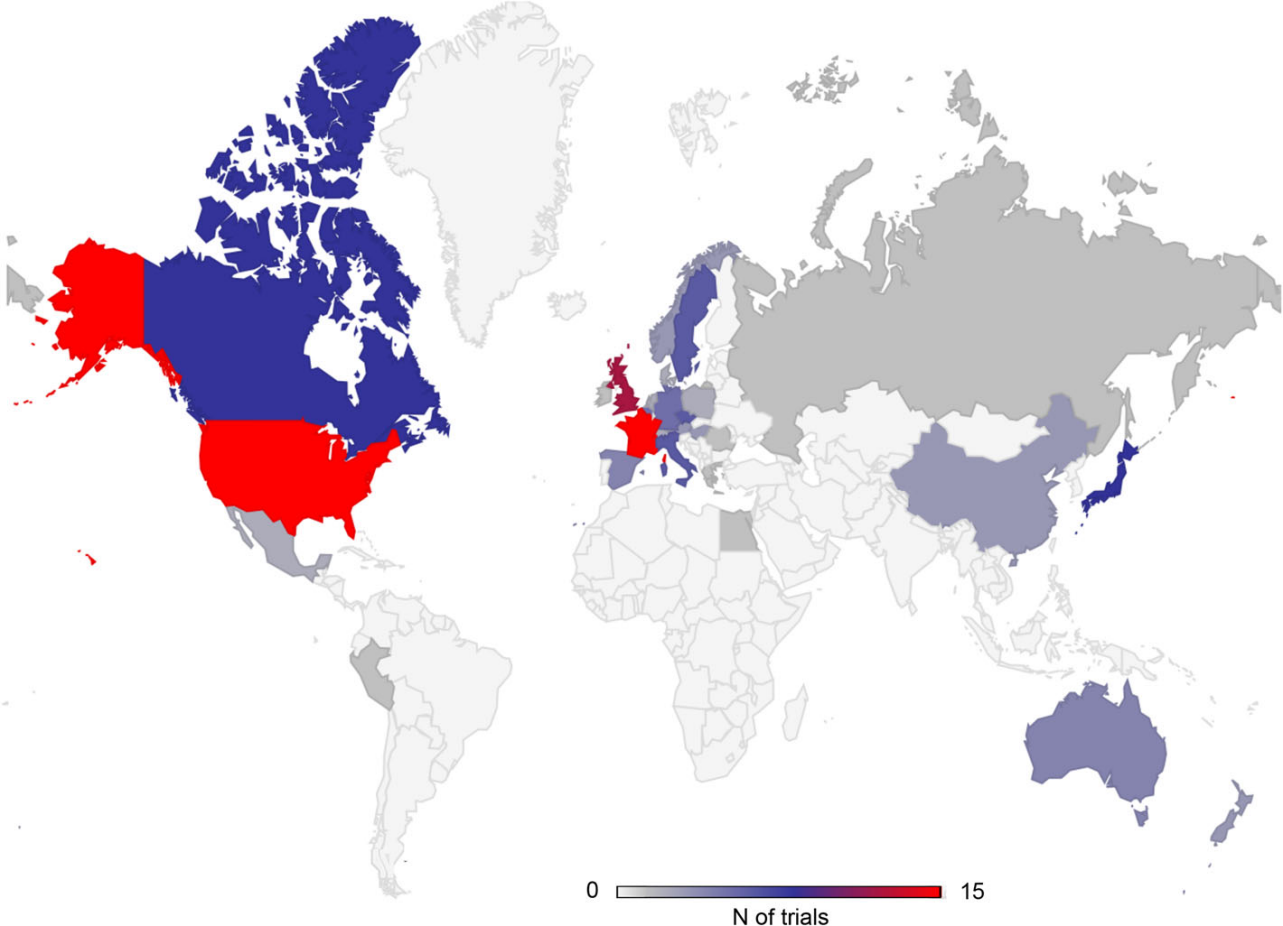
Shows the countries where AAV-RCTs were (or planned to be) conducted

Among the 40 RCTs identified (flow-chart in online appendix), 22 (38.5%) were conducted in Europe, 15 (26.5%) in North America (Fig. 1). Twenty-nine (72,5%) were conducted in a single-country (9 in France, 8 in US, 4 in Japan, 3 in China, 3 in UK, 1 in Egypt, 1 in Czech Republic).

The RCTs retrieved were mostly parallel arm (*n* = 38; 95%), late development phases (phase II/III, III, IV) (*n* = 27; 67.5%), non-industry funded studies (*n* = 26; 65%) planning to enrol a median of 68 (IQR 36–138) patients. Seventeen (42.5%), and 2 (5%) RCTs planned to enroll more than 100 and 200 patients, respectively. Among the 5 studies with available results, only one enrolled a lower number of patients than originally planned. Table 1 shows the main features of the RCTs included. The complete list of the trials included is in online appendix.

**Table 1:** Characteristics of RCTs in AAV patients from WHO International Clinical Trials Registry Platform (ICTRP)

**Item and subcategory**	**RCTs**
	*N* = 40
**Single-country studies**	29 (72.5)
**Location of studies (continent)**^**a**^	
Europe	22 (38.5)
North America	15 (26.5)
Asia	12 (21)
South America	2 (3)
Oceania	4 (7)
Africa	1 (2)
Unclear	1 (2)
**Type of intervention**	
**Pharmacological therapy**	38 (95)
Non-biological	22 (55)
Biological	16 (40)
**Study design**	
Parallel group	38 (95)
Crossover	2 (5)
**Type of comparator**	
Active (pharmacologic)	22 (55)
Placebo	14 (35)
Usual care	3 (7.5)
No intervention	1 (2.5)
**Phase of development**	
Early development (phase 0, I)	0 (0)
Middle development (phase I/II, II)	10 (25)
Late development (phase II/III,III, IV)	27 (67.5)
Not reported/not applicable	3 (7.5)
**Status of recruitment**	
Closed recruitment (completed recruitment or terminated studies)	14 (35)
Recruiting/ongoing	14 (35)
Not yet recruiting	7 (17.5)
Withdrawn	3 (7.5)
Unknown	2 (5)
**Industry-funded**	14 (35)
**Sample size**	
No. of patients planned to be included per study	68 (36–138)
(median, IQR)	range 14–704

^a^Multiple answers were possible. *RCTs* randomized controlled trials

### Eligible criteria, population targeted

In most RCTs (*n* = 37; 92.5%), patients aged > 65 years were allowed to be included. Criteria to identify diseases were mostly the original or revised Chapel-Hill nomenclature or ACR Criteria (*n* = 17; 42.5%). Half of studies (*n* = 20; 50%) were designed to investigate GPA and MPA ± renal-limited AAV, 5 (12.5%) GPA and MPA and EGPA ± renal-limited patients, whereas 11 (27.5%) planned to include a single disease: 6 (15%) GPA, 3 (7.5%) EGPA and 2 (5%) MPA.

ANCA positivity was a mandatory eligibility criterion in about 40% of studies. In detail, 8 studies (20%) required a positive test for anti-MPO or anti-PR3; 2 (5%) a positive test for either anti-MPO or anti-PR3 antibodies or for ANCA by immunofluorescence; one RCT, a positive ANCA test by immunofluorescence; in 4 (10%) studies the test to be used to detect ANCA antibodies was not specified. Trials requiring a positive ANCA test enrolled GPA and MPA ± renal-limited (*n* = 11); GPA and MPA and EGPA ± renal-limited (*n* = 4). There was no trial on a single vasculitis restricting the enrolment to ANCA positive patients. Table 2 summarizes the main features of the population included in RCTs.

**Table 2:** Features of population included in RCTs

**Population**	***N*** **= 40**
**Age**	
Adults and seniors (adults > 65 years)	33 (82.5)
Adults (18 to 65 years)	2 (5)
All ages	4 (10)
Children	1 (2.5)
**Disease**	
GPA + MPA (± renal limited AAV)	20 (50)
only GPA	6 (15)
GPA + MPA + EGPA (± renal limited AAV)	5 (12.5)
only EGPA	3 (7.5)
only MPA	2 (5)
AAV with other vasculitides/autoimmune diseases	4 (10)
**Diagnosis status**	
Newly diagnosed and/or prevalent patients	31 (77.5)
Prevalent patients	6 (15)
Newly diagnosed patients	3 (7.5)
**Classification criteria used to include patients**	
Chapel-Ill nomenclature	6 (15)
Revised Chapel-Ill nomenclature	3 (7.5)
ACR criteria	5 (12.5)
Chapel-Hill nomenclature and/or ACR criteria ± other	3 (7.5)
Clinical diagnosis or not specified	19 (47.5)
Other	4 (10)
**Main treatment indication**	
Induction of remission	16 (40)
Maintenance of remission	13 (32.5)
Induction and maintenance of remission	4 (10)
Other	7 (17.5)
**Classification of important outcomes**	
Patients important outcomes (PIO)	37/43* (85)
Surrogate outcomes (SO)	6/43*
No. of studies with at least one PIO as primary outcome	33 (82.5)
**Glucocorticoids dose/use included in primary outcome**	11/43* (25%)
Glucocorticoids dose/use as the only primary outcome	2/43*

If not otherwise specified, data are expressed as number (percentages). *Multiple answers were possible, *GPA* Granulomatosis with polyangiitis, *MPA* Microscopic polyangiitis, EGPA Eosinophilic granulomatosis with polyangiitis, *AAV* ANCA-associated vasculitis, PO primary outcome. * Forty-three primary outcomes for the 40 studies retrieved

### Interventions investigated and main study outcomes

The interventions consisted in pharmacologic treatments for most of trials (*n* = 38; 95%); procedures (i.e. plasma exchange/double filtration plasmapheresis) were tested in 2 studies.

**Fig. 2:**
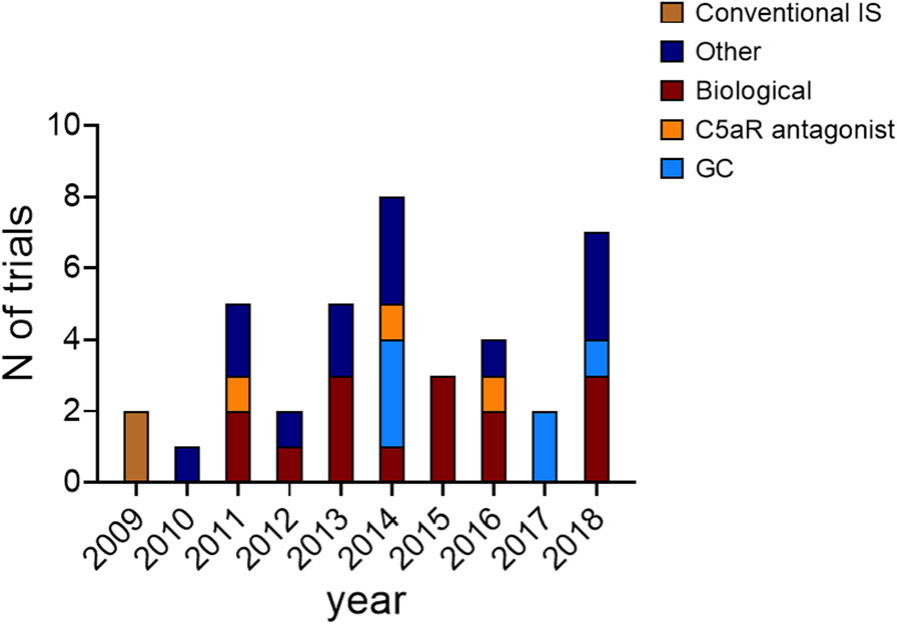
Shows the evolution over time of class of drug investigated. IS. Immunosuppressors; C5aR. C5a receptor; GC. Glucocorticoids

Main study objectives were the evaluation of treatment efficacy to induce (*n* = 16; 40%), maintain (*n* = 13; 32.5%) or induce/maintain (*n* = 4; 10%) disease remission. Among pharmacologic interventions, monoclonal antibodies (n = 16), GC (*n* = 5), complement antagonist (*n* = 3), and conventional immunosuppressors (n = 3) were the main classes of drug investigated. The remaining studies evaluated the utility of giving valaciclovir to reduce CMV reactivation in AAV patients receiving immunosuppressors; the role of statin in preventing atherosclerosis; the efficacy of pneumococcal vaccination; the influence of endothelin antagonists on vascular response; the utility of biomarkers to assess response to treatment. Figure 2 shows the time trend of the class of drugs investigated.

**Fig. 3:**
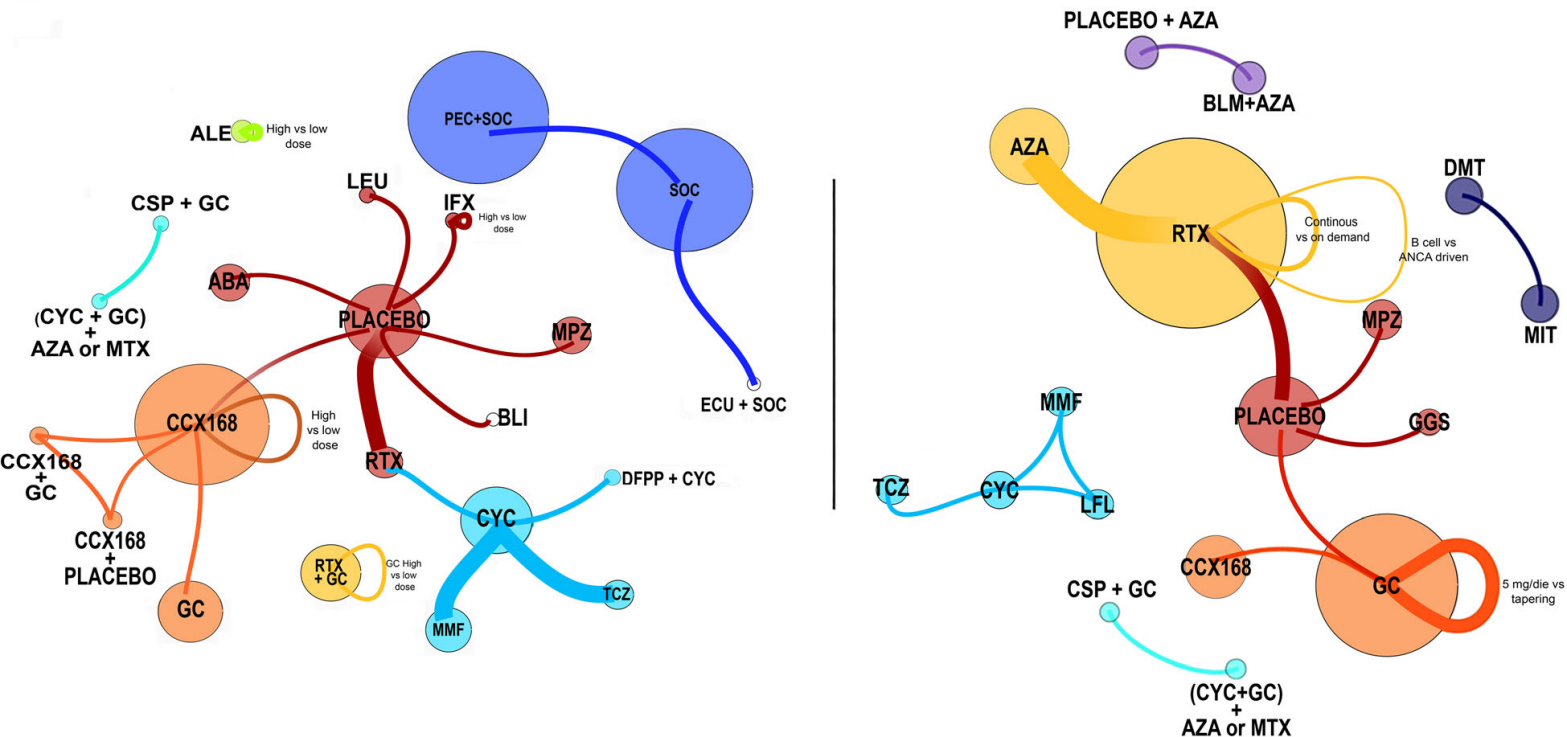
Networks of RCTs investigating interventions to induce (left) or maintain (right) disease remission. Each circle represents an intervention (arm). The dimension of the circle is proportional to the number of patients enrolled/planned to be enrolled in that arm. Two or more interventions are connected when compared within a trial. The thickness of the connector is proportional to the number of trials planned for each comparison. List of abbreviations for treatments included in networks. ABA, Abatacept; ALE, Alemtuzumab; AZA, Azathioprine; BLI, Blisibimod; BLM, Belimumab; CCX168, Avacopan; CYC, Cyclophosphamide; DFPP + CYC, Double filtration plasmapheresis + cyclophosphamide; DMT, Discontinuation of maintenance treatment; ECU, Eculizumab; GC, Glucocorticoids; GGS, Freeze-Dried Sulfonated Human Normal Immunoglobulin; CSP, Gusperimus; IFX, IFX-1 CaCP 29; LEU, Depot leuprolide acetate; LFL, Leflunomide; MIT, Maintenance of immunosuppressive treatment; MMF, Mycophenolate mofetil; MPZ, Mepolizumab; MTX, Methotrexate; PEC, Plasma exchange; RTX, Rituximab; SOC, Standard of care

Comparators more frequently used were an active pharmacologic treatment (*n* = 22; 55%) or placebo (*n* = 14; 35%). Figure 3 summarizes the RCTs investigating interventions given to induce or maintain disease remission.

### Classification and description of primary outcomes

In 7 (17.5%) RCTs the primary outcome was not considered “patient important” (PIO). When focusing only on larger trials, the percentage of PIO among the primary outcome was of 88% (*n* = 15) for studies with more than 100 patients. In 11 (25%) cases, the use or dose of GC was part of the primary outcome.

Thirty-eight (95%) RCTs had an efficacy primary endpoint, being remission in 16 (40%), and relapse in 13 (32.5%). The definition of remission included the Birmingham Vasculitis Activity Score (BVAS) [19] in most of cases (*n* = 9/16; 56%), followed by BVAS version 3 [20] (*n* = 3/16; 19%), BVAS for Wegener’s Granulomatosis (BVAS/WG) [21] (*n* = 2/16; 12.5%) and other definitions (*n* = 2/16; 12.5%). The use of GC was part of remission definition in 9 primary outcomes (4 for EGPA, 4 for GPA/MPA, 1 for GPA), with different minimal daily doses required (< 10 mg to drug discontinuation). In studies enrolling GPA and MPA ± renal-limited vasculitis, and having remission as primary outcome, GC use was not included in the definition of remission in 5/9 studies, while the achievement of a daily dose < 10 mg, or ≤ 7.5 mg, GC discontinuation or adherence to GC tapering was requested in the remaining 4 trials. In the 3 EGPA-related trials, the use of GC was always part of remission definition (in 2 if a dose ≤7.5 mg/day, in one if ≤4 mg/day was achieved). Finally, in 3 studies aiming to evaluate remission in GPA patients, GC use was not mentioned in two RCTs or needed to be ≤10 mg/day in the other study.

Relapse was defined by using BVAS (*n* = 4), BVAS/WG (*n* = 3), BVASv3 (*n* = 1), and decision to increase GC (n = 1) (definition was not provided in 4 studies). One study had a patient reported outcome (PRO), the Patient-Reported Outcomes Measurement Information System (PROMIS) Global Physical Health as primary outcome.

### Evolution over time of the main characteristics of the clinical trials

**Fig. 4:**
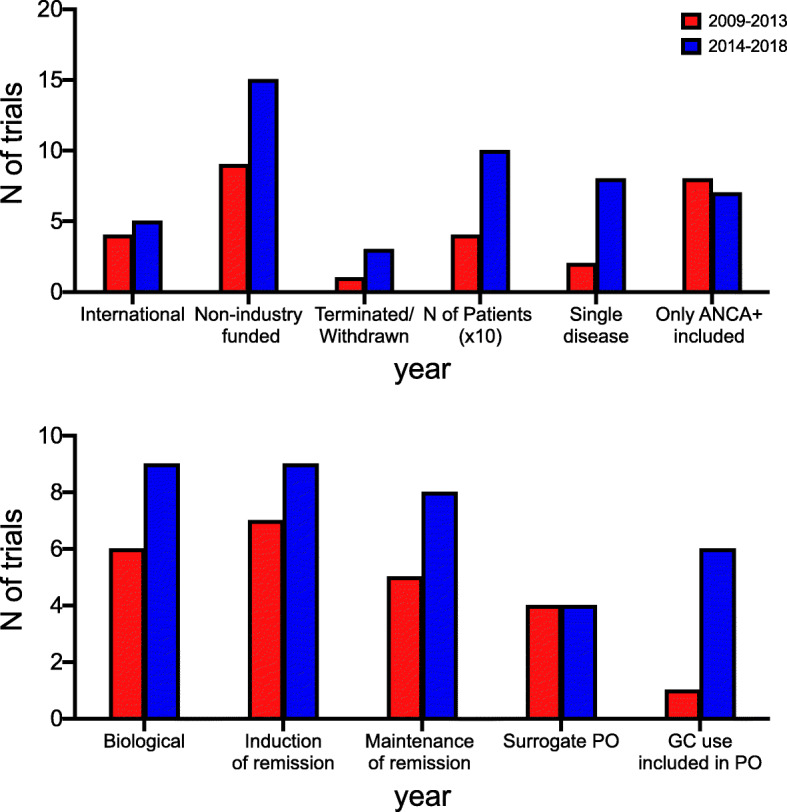
Main RCTs features in 2009–2013 and 2014–2018

Figure 4 shows the main characteristics of the trials starting in 2009–2013 in comparison to those that started in 2014–2018. As compared to the former period, between 2014 and 2018 we recorded a higher number of trials including a single disease (8 vs 2), not funded by industry (15 vs 9), investigating biological treatments (9 vs 6), having GC use/dose as primary outcome (6 vs 1). Moreover, there was also a trend towards a higher number of patients planned to be enrolled in 2014–2018 [median 98 (42–140) vs 40 (IQR 14–106)]. The number of trials targeting a single disease increased from 2 (2 MPA) in 2009–2013 to 8 (5 GPA, 3 EGPA) in 2013–2018. Studies on single diseases were more likely to enrol patients independently from their ANCA status (11/11 vs 14/29 ANCA positivity not required; *p* = 0.002), and to be conducted in a single country (8/11 vs 7/29; *p* = 0.009) if compared to trials enrolling more than one disease. No other difference was identified (data not shown).

#### Withdrawn/terminated studies: prevalence and reasons

Three (7.5%) RCTs were withdrawn and 4 (10%) ended prematurely (‘terminated’) before completion. Withdrawn was reported to be due to: no eligible patient to be enrolled (2 study); unknown reason (1 study). Reasons for early termination were: slow recruitment (2 studies), change of design consideration (1 study), unknown reason (1 study). Withdrawn or terminated studies were mostly two parallel arm trials investigating pharmacologic treatments (*n* = 6/7; 86%) given to induce disease remission (*n* = 5/7; 71%) and enrolling more than one vasculitis (n = 5; the remaining 2 planned to include only GPA patients). Six of them (86%) were intended to be conducted in a single country, 4 (57%) were industry funded, 4 (57%) required ANCA positive patients. No difference was found in main study features between withdrawn/terminated studies and ongoing, still recruiting or completed RCTs (online appendix file).

## Discussion

RCTs conducted in AAV in the last decade were mainly single-country, two parallel arms, late development studies investigating the efficacy of pharmacological treatments to induce or maintain remission. Most of the studies were designed to include more than one disease, and often targeted ANCA-positive patients. The majority of primary outcomes were considered to be patient-important, but definitions of disease states such as remission were heterogeneous, and only one primary outcome was a PRO. The number of RCTs investigating a single disease, not funded by industry, incorporating GC use in primary outcomes, together with the expected sample size increased over time.

Although building evidence for rare diseases is challenging, RCTs conducted in last years have contributed to greatly improve the management of AAV [22–25]. More efficacious and less toxic treatment strategies have become available in daily practice. The rituximab, a chimeric monoclonal antibody against the protein CD20, has been shown to be a valid alternative to cyclophosphamide to induce remission [25], and it is today the first-choice for maintaining remission in GPA and MPA [23]. The study PEXIVAS, whose results have been recently published, has questioned the utility to perform plasma exchange in severe AAV patients [26]. Additionally, different trials have provided further knowledge on the best duration of maintenance treatment, and more studies have been specifically designed for EGPA, the rarest among the AAV. A large randomized trial has demonstrated the utility of the mepolizumab (an anti-IL-5 recombinant humanized monoclonal antibody) for severe and refractory EGPA, and as corticosteroid-sparing agent [27]. Rituximab is also being studied in EGPA.

Aware of these important results, we planned the present study with the aim to describe the main features of AAV-RCTs, and identify potential room for improvement for trial design. Differences in eligibility criteria and in the number of diseases investigated per trial, discrepancies in definition of important disease states like remission, are relevant points that deserve to be discussed.

Patient samples included in RCTs were quite heterogeneous. First, in most cases more than one disease was investigated. Although this represents the obvious solution to get a needed sample size in such rare diseases, differences in clinical presentation and evolution among AAV could unfortunately hamper the translation of study results in clinical setting. Moreover, disease definition relied on different criteria, among which ACR criteria and/or Chapel-Hill nomenclature were used in about half of studies, whereas in the other half, only a clinical diagnosis was required. Additionally, the set of trials retrieved was split in almost two equal-sized groups of ANCA positive and mixed ANCA positive and negative patients. This means that slightly less than half of studies did not incorporate ANCA negative patients who represent about 10–20% of GPA [28], and up to 70% of EGPA population [7], with a consequent obvious impaired generalizability of study conclusions.

The majority of study outcomes was considered to be ‘patient-important’. Most of trials were designed to assess the efficacy of treatments given to induce or maintain remission, or their safety, which are undoubtedly very important outcomes and of utmost interest for the clinicians. The choice of ‘patient-important’ outcomes has been recognized as a priority to avoid waste of time and resources, and represents a successful achievement in the field [29]. However, the definitions of important outcomes - such as remission - were inconsistent across the studies. For example, original BVAS or later versions were nearly always used to rule out the occurrence of an active disease on a clinical basis, but GC use was not systematically incorporated in the definition of remission, or different minimal GC doses were required. This contrasts with EULAR guidelines [11] which recommended to define remission taking also into account the allowable dose or dose range of GC, and the period during which such dose should be kept stable. The lack of homogeneous definitions impairs inter-study comparison. This point would hopefully deserve to be object of further research.

Finally, in only one trial a patient-reported outcome was chosen as primary study endpoint. Efforts are needed to incorporate needs and perspectives of patients in main study outcomes [30].

This study has some limitations. First, we could have missed some clinical trials whose protocol had not been registered in online platforms. Second, some important study features (for example details on diagnostic or classification criteria) could have been provided only in final publications, and not in online databases, and consequently not analyzed in our study.

## Conclusions

In conclusion, a higher number of trials (overall and targeting single diseases) with an increasing sample size have been designed and conducted in the last decade, and have tremendously contributed to improve the care of AAV patients. A better harmonization of eligibility, and outcome criteria across studies is an important objective to pursue in next future.

## Data Availability

The dataset used during the current study is available from the corresponding author on reasonable request.

## Abbreviations

AAV: ANCA-associated vasculitis
ANCA: Antineutrophil cytoplasmic antibodies (ANCA)-associated vasculitis
EGPA: Eosinophilic granulomatosis with polyangiitis
GPA: Granulomatosis with polyangiitis
MPA: Microscopic polyangiitis
PO: Primary outcome
RCTs: Randomized controlled trials

## References

[1] Watts RA, Robson J (2018). Introduction, epidemiology and classification of vasculitis. Best Pract Res Clin Rheumatol.

[2] Jennette JC, Falk RJ, Bacon PA, Basu N, Cid MC, Ferrario F (2013). 2012 revised international Chapel Hill consensus conference nomenclature of Vasculitides. Arthritis Rheum.

[3] Millet A, Pederzoli-Ribeil M, Guillevin L, Witko-Sarsat V, Mouthon L (2013). Antineutrophil cytoplasmic antibody-associated vasculitides: is it time to split up the group?. Ann Rheum Dis.

[4] Jayne D, Rasmussen N, Andrassy K, Bacon P, Tervaert JW, Dadoniené J (2003). A randomized trial of maintenance therapy for vasculitis associated with antineutrophil cytoplasmic autoantibodies. N Engl J Med.

[5] Aouba A, Pagnoux C, Bienvenu B, Mahr A, Guillevin L (2008). Analysis of Wegener's granulomatosis responses to rituximab: current evidence and therapeutic prospects. Clin Rev Allergy Immunol.

[6] Sinico RA, Di Toma L, Maggiore U, Bottero P, Radice A, Tosoni C (2005). Prevalence and clinical significance of antineutrophil cytoplasmic antibodies in Churg-Strauss syndrome. Arthritis Rheum.

[7] Comarmond C, Pagnoux C, Khellaf M, Cordier JF, Hamidou M, Viallard JF (2013). Eosinophilic granulomatosis with polyangiitis (Churg-Strauss): clinical characteristics and long-term followup of the 383 patients enrolled in the French Vasculitis study group cohort. Arthritis Rheum.

[8] Jardel S, Puéchal X, Le Quellec A, Pagnoux C, Hamidou M, Maurier F (2018). Mortality in systemic necrotizing vasculitides: a retrospective analysis of the French Vasculitis study group registry. Autoimmun Rev.

[9] Puéchal X (2019). Targeted immunotherapy strategies in ANCA-associated vasculitis. Joint Bone Spine.

[10] Mukhtyar C, Flossmann O, Hellmich B, Bacon P, Cid M, Cohen-Tervaert JW (2008). Outcomes from studies of antineutrophil cytoplasm antibody associated vasculitis: a systematic review by the European league against rheumatism systemic vasculitis task force. Ann Rheum Dis.

[11] Hellmich B, Flossmann O, Gross WL, Bacon P, Cohen-Tervaert JW, Guillevin L (2007). EULAR recommendations for conducting clinical studies and/or clinical trials in systemic vasculitis: focus on anti-neutrophil cytoplasm antibody-associated vasculitis. Ann Rheum Dis.

[12] WHO. International Clinical Trials Registry Platform. Available: http://apps.who.int/trialsearch/. Accessed 17 Jan 2019.

[13] DrugBank (version 5.1.0, released 2018-04-02). Available: https://www.drugbank.ca/. Accessed 29 Apr 2019.

[14] Jennette JC, Falk RJ, Andrassy K, Bacon PA, Churg J, Gross WL (1994). Nomenclature of systemic vasculitides. Proposal of an international consensus conference. Arthritis Rheum.

[15] Leavitt RY, Fauci AS, Bloch DA, Michel BA, Hunder GG, Arend WP (1990). The American College of Rheumatology 1990 criteria for the classification of Wegener’s granulomatosis. Arthritis Rheum.

[16] Masi AT, Hunder GG, Lie JT, Michel BA, Bloch DA, Arend WP (1990). The American College of Rheumatology 1990 criteria for the classification of Churg-Strauss syndrome (allergic granulomatosis and angiitis). Arthritis Rheum.

[17] Ferreira-González I, Busse JW, Heels-Ansdell D, Montori VM, Akl EA, Bryant DM (2007). Problems with use of composite end points in cardiovascular trials: systematic review of randomised controlled trials. BMJ.

[18] Gandhi GY, Murad MH, Fujiyoshi A, Mullan RJ, Flynn DN, Elamin MB (2008). Patient-important outcomes in registered diabetes trials. JAMA.

[19] Luqmani RA, Bacon PA, Moots RJ, Janssen BA, Pall A, Emery P (1994). Birmingham Vasculitis activity score (BVAS) in systemic necrotizing vasculitis. QJM.

[20] Mukhtyar C, Lee R, Brown D, Carruthers D, Dasgupta B, Dubey S (2009). Modification and validation of the Birmingham Vasculitis activity score (version 3). Ann Rheum Dis.

[21] Stone JH, Hoffman GS, Merkel PA, Min YI, Uhlfelder ML, Hellmann DB (2001). A disease-specific activity index for Wegener’s granulomatosis: modification of the Birmingham Vasculitis activity score. International network for the study of the systemic Vasculitides (INSSYS). Arthritis Rheum.

[22] Tarzi RM, Mason JC, Pusey CD (2014). Issues in trial design for ANCA-associated and large-vessel vasculitis. Nat Rev Rheumatol.

[23] Guillevin L, Pagnoux C, Karras A, Khouatra C, Aumaître O, Cohen P (2014). Rituximab versus azathioprine for maintenance in ANCA-associated vasculitis. N Engl J Med.

[24] Puéchal X, Pagnoux C, Perrodeau É, Hamidou M, Boffa JJ, Kyndt X (2016). Long-term outcomes among participants in the WEGENT trial of remission-maintenance therapy for granulomatosis with polyangiitis (Wegener's) or microscopic polyangiitis. Arthritis Rheumatol.

[25] Specks U, Merkel PA, Seo P, Spiera R, Langford CA, Hoffman GS (2013). Efficacy of remission-induction regimens for ANCA-associated vasculitis. N Engl J Med.

[26] Walsh M, Merkel PA, Peh CA, Szpirt WM, Puéchal X, Fujimoto S (2020). Plasma exchange and glucocorticoids in severe ANCA-associated Vasculitis. N Engl J Med.

[27] Wechsler ME, Akuthota P, Jayne D, Khoury P, Klion A, Langford CA (2017). Mepolizumab or placebo for Eosinophilic Granulomatosis with Polyangiitis. N Engl J Med.

[28] Terrier B, Dechartres A, Deligny C, Godmer P, Charles P, Hayem G (2017). Granulomatosis with polyangiitis according to geographic origin and ethnicity: clinical-biological presentation and outcome in a French population. Rheumatology (Oxford).

[29] Yordanov Y, Dechartres A, Atal I, Tran VT, Boutron I, Crequit P (2018). Avoidable waste of research related to outcome planning and reporting in clinical trials. BMC Med.

[30] Milman Nataliya, McConville Eilish, Robson Joanna C., Boonen Annelies, Tugwell Peter, Wells George A., Chaudhuri Dipayan, Dawson Jill, Tomasson Gunnar, Ashdown Susan, Gebhart Don, Lanier Georgia, Peck Jacqueline, McAlear Carol A., Kellom Katherine S., Cronholm Peter F., Merkel Peter A. (2019). Updating OMERACT Core Set of Domains for ANCA-associated Vasculitis: Patient Perspective Using the International Classification of Function, Disability, and Health. The Journal of Rheumatology.

